# Infrequent detection of human papillomavirus infection in head and neck cancers in the Central African Republic: a retrospective study

**DOI:** 10.1186/s13027-019-0225-x

**Published:** 2019-04-08

**Authors:** Boniface Kofi, Christian Diamant Mossoro-Kpinde, Ralph-Sydney Mboumba Bouassa, Hélène Péré, Leman Robin, Gérard Gresenguet, Laurent Bélec

**Affiliations:** 1 Laboratoire National de Biologie Clinique et de Santé Publique, Bangui, Central African Republic; 2 Faculté des Sciences de la Santé, Université de Bangui, Bangui, Central African Republic; 3 Ecole Doctorale d’Infectiologie Tropicale, Franceville, Gabon; 4 Assistance Publique-Hôpitaux de Paris, Hôpital Européen Georges Pompidou, Laboratoire de Virologie, and Faculté de Médecine Paris Descartes, Université Paris V, Paris Sorbonne Cité, Paris, France; 5 Unité de Recherches et d’Intervention sur les Maladies Sexuellement Transmissibles et le SIDA, et Faculté des Sciences de la Santé de Bangui, Bangui, Central African Republic

**Keywords:** HPV, Oropharynx, Oral cavity, Central Africa, Vaccination

## Abstract

We carried out a retrospective study on the prevalence of HPV and genotype distribution by nested PCR and nucleotide sequencing analysis, in formalin-fixed, paraffin-embedded biopsies of 135 head and neck cancers (HNC) and 29 cervical cancers received between 2009 and 2017 for diagnosis at the Laboratoire National de Biologie Clinique et de Santé Publique of Bangui, the capital city of the Central African Republic. One oropharyngeal squamous cell carcinoma sample was positive for HPV type 16. The overall HPV prevalence in HNC biopsies was 0.74% (95% CI: 0.0–2.2). Among the 29 cervical cancer samples, 19 (65.5%; 95% CI: 48.2–82.8) were positive for HPV. These results indicate that HNC are infrequently associated with HPV infection in the Central African Republic.

Head and neck cancers (HNC), excluding nasopharyngeal, most frequently squamous cell carcinoma, are among the most aggressive tumours worldwide [1]. In addition to alcohol and tobacco consumption, human papillomaviruses (HPV) have shown to be associated with the development of a significant proportion of oropharyngeal cancers [2]. Since 2007, HPV is considered as an independent risk factor for head and neck squamous cell carcinoma (HNSCC) by the World Health Organization (WHO)‘s International Agency for Research on Cancer (WHO/IARC) [2]. Recently, the “4th Edition of the WHO Classification of Head and Neck Tumours: Oropharynx”, classifies the squamous cell carcinoma of the oropharynx (OPSCC) on the basis of HPV status [3]. HPV-positive OPSCC constitutes a tumor entity with a distinct epidemiological profile, with specific genetic features, clinical presentations and outcomes.

Although HPV contribution to HNC is substantial, high heterogeneity by cancer site, region and sex has been reported [4]. Population-based cancer registries have demonstrated a clear pattern of increasing incidence of HPV-related OPSCC in developed countries across multiple continents. However, here is a paucity of knowledge on the role of HPV in HNC in the majority of developing countries, including in Africa [5]. A recent study in West Africa (Senegal) reported a very low prevalence (3.4%) of HPV in HNC biopsies, suggesting no significant association with HPV infection [6]. Similarly, two other studies from Mozambique and Nigeria failed to detect HPV DNA in HNC biopsies [7, 8].In South Africa, the overall incidence for HNC declined in the general population from 1994 to 2013, but the relationship of HNC and HPV was not investigated [9]. In Middle Africa, the incidence of HNC was estimated nearly two-fold lower than the worldwide rate (4.5 versus 8.0 per 100,000 new cases/year), while an unexpected high incidence of HNC (9.4) was recently reported in Gabon [10]. In the context of high prevalence of oncogenic HPV and HPV-related cervical cancer in Gabon, the possibility of increasing incidence of HPV-related HNC has been hypothesized, but not yet demonstrated [11].

The aim of our research was to provide original data on the role of HPV in invasive HNC in the Central African Republic, a country of Middle Africa, where cervical cancer is the second most frequent cancer in women between 15 and 44 years of age, suggesting high prevalences of oncogenic HPV among general population [12]. The crude incidence rates of HNC were estimated in 2015 at 1.2 in males and 0.5 in females per 100,000 and per year [8].

The *Laboratoire National de Biologie Clinique et de Santé Publique* (“LNBCSP”) of Bangui, the capital city of the Central African Republic, is the reference national laboratory in laboratory medicine, including pathology laboratory. The LNBCSP received all biospies and other pathological investigations for the whole country, and also implement the cancer registration.

During 2009 to 2017, the LNBCSP received 135 invasive biopsies from HNC (Table 1). For each specimen, routine histological examination on hematoxylin-eosin stained slide prepared from a formalin-fixed, paraffin-embedded (FFPE) biopsy, confirmed invasive diagnosis.

**Table 1:** HPV DNA detection in 135 head and neck cancer cases from oral cavity (*n* = 45), nasopharynx (*n* = 9), oropharynx (*n* = 19), pharynx unspecified (*n* = 25) and larynx (*n* = 37) in Central African Republic, by patients’ characteristics during the period 2009 to 2017

Variables	Samples tested for HPV		HPV positive sample	
	N	%	N	% (95% CI)
Gender				
Male	74	54.8	1^a^	1.35 (0.0–3.9)
Female	61	45.2	0	0.0 (0.0–0.06)^b^
Age at diagnosis (years)				
≤39	27	20.0	0	0.0 (0.0–0.13)^b^
40-49	17	12.6	0	0.0 (0.0–0.18)^b^
50-59	15	11.1	1	6.67 (0.0–19.3)
≥60	76	56.3	0	0.0 (0.0–0.05)^b^
Period of diagnosis				
2009–2011	32	23.7	0	0.0 (0.0–0.11)^b^
2012-2014	64	47.4	1	1.56 (0.0–4.6)
2015–2017	39	28.9	0	0.0 (0.0–0.09)^b^
Histological type				
Squamous cell carcinoma	129	95.6	1	0.78 (0.0–2.3)
Adenocarcinoma	6	4.4	0	0.0 (0.0–0.39)^b^

^a^Positivity for HPV-16;

^b^One-sided, 97.5% confidence interval

*CI* Confidence interval

Afterwards, a paraffin tissue 5- to 20- μm sections was subjected to virological analysis into the ISO 15189-accredited virology laboratory of the Hôpital européen Georges Pompidou, Paris, France. Indeed, FFPE biopsy samples are frequently the only available ones for molecular testing after pathological examination. However, FFPE samples necessitate specific processing before PCR analysis because formalin fixation induces fragmentation of nucleic acids. Thus, the sample was treated with 250 μl of freshly prepared proteinase K solution to extract and purify DNA onto a silicate column (QIAmp DNA mini kit, Qiagen, Courtaboeuf, France), following manufacturer’s instructions. Finally, the total DNA was eluted with 100 μl of DNase-free elution buffer.. The amplification of albumin DNA by in house real-time PCR was used as marker of DNA integrity. Finally, a 1 μg amount of extracted DNA was amplified by “hot start” PCR with degenerate HPV consensus primers MY09 and MY11, as described previously [13], followed by nested PCR using the GP5+ and GP6+ primers, as previously described [13].

FFPE biopsies of 25 squamous cell carcinoma and 4 adenocarcinoma of the cervix were also analyzed in parallel. Caskie cells (ATCC® CRM-CRL-1550) were used to obtain HPV DNA-positive control. Each isolate was sequenced in both directions. HPV genotypes were determined by direct sequencing of PCR products. Briefly, amplicons were subjected to cycle sequencing with the ABI PRISM Big Dye Terminator Cycle Sequencing Ready Reaction Kit (Applied Biosystems Foster City, California, USA). The sequencing reactions were then run on the ABI Prism 3700 Genetic Analyzer (Applied Biosystems). All clinical sequences were submitted to the BLAST server (https://blast.ncbi.nlm.nih.gov/Blast.cgi) to be aligned and matched with all HPV sequences available within this database. HPV type was identified on the basis of ≥95% sequence homology in L1 region from HPV sequences available in HPV online databases. The assigned genotype was further confirmed by phylogenetic assay using Mega 2.1 software (www.megasoftware.net).

All DNA samples were positive for albumin detection confirming the good quality of extracted DNA and the possibility to further detect HPV DNA. Among the HNC biopsies, only one oropharyngeal squamous cell carcinoma biopsy sample was positive for HPV-16. The overall HPV prevalence in the HNC biopsy series during the whole study period was 0.74% (95% CI: 0.0–2.2) (Table 1). Caskie cells were positive for HPV-16. Among the 29 cervical cancer biopsy samples, 19 (65.5%; 95% CI: 48.2–82.8) were positive for HPV [HPV-16: *n* = 11, HPV-18: *n* = 2, HPV-31: *n* = 3, HPV-33: n = 2, HPV-69: n = 1], including 17 squamous cell carcinoma and 2 adenocarcinoma.

To our knowledge, this is the first study on the presence of HPV DNA in head and neck tumors in Central Africa. During a ten years retrospective survey, HPV DNA could be detected in only one out of 135 (less than 1%) biopsy samples from patients suffering from HNC, suggesting that HPV prevalence in HNC in the Central African Republic is very infrequent. In contrast, the majority (65.5%) of cervical cancer biopsy samples collected and analyzed under the same conditions were positive for high-risk HPV.

Our observations appear very different from what has been reported previously in the other regions of the world, where HPV DNA can be detected overall in 26% of HNC [14]. Similar low HPV prevalences in HNC have been previously reported in the African-American community in the USA [15] and in Senegal [5]. Taken together, the low HPV prevalence in invasive HNC in Central African Republic suggests that other established risk factors such as alcohol and tobacco consumption as well as eating habits may play a more significant etiological role than HPV infection in HNC in this country. Our findings need to be further validated with supplementary studies that include larger case series and the assessment of region-specific risk factors.

Our study has limitations. The retrospective analysis and the small size of biopsy specimens may have introduced selection bias. In addition, 60.7% of study specimens were HNC from oral cavity or larynx, which are driven more by alcohol and smoking than HPV [4]. Furthermore, HPV prevalence rate in cervical cancer biopsies appear lower than that reported in other studies [16], suggesting possible low detection sensitivity due to the storage protocol of biopsy samples into paraffin, as mentioned elsewhere [17].

## Abbreviations

BLAST: Basic local alignment search tool
DNA: Deoxyribonucleic acid
FFPE: Formalin-fixed, paraffin-embedded
HNC: Head and neck cancer
HNSCC: Head and neck squamous cell carcinoma
HPV: Human papillomavirus
IARC: International Agency for Research on Cancer
ISO: International Organization for Standardization
L1: Late protein 1
LNBCSP: Laboratoire National de Biologie Clinique et de Santé Publique
OPSCC: Oropharyngeal squamous cell carcinoma
PCR: Polymerase chain reaction
WHO: World health organization.

## References

[1] Ferlay J, Soerjomataram I, Dikshit R, Eser S, Mathers C, Rebelo M, Parkin DM, Forman D, Bray F (2015). Cancer incidence and mortality worldwide: sources, methods and major patterns in GLOBOCAN 2012. Int J Cancer.

[2] International Agency for Research on Cancer, 2010 (2010). Monographs on the evaluation of carcinogenic risks to humans, vol. 100. A review of human carcinogens. Part B: biological agents.

[3] EI-Naggar AK, Chan JKC, Grandis JR, Takata T, Slootweg PJ (2017). WHO classification of head and neck Tumours (4th edition).

[4] Castellsagué X, Alemany L, Quer M, Halec G, Quirós B, Tous S, Clavero O, Alòs L, Biegner T, Szafarowski T, Alejo M, Holzinger D, Cadena E, Claros E, Hall G, Laco J, Poljak M, Benevolo M, Kasamatsu E, Mehanna H, Ndiaye C, Guimerà N, Lloveras B, León X, Ruiz-Cabezas JC, Alvarado-Cabrero I, Kang CS, Oh JK, Garcia-Rojo M, Iljazovic E, Duarte F, Nessa A, Tinoco L, Duran-Padilla MA, Pirog EC, Viarheichyk H, Morales H, Costes V, Félix A, Germar MJ, Mena M, Ruacan A, Jain A, Mehrotra R, Goodman MT, Lombardi LE, Ferrera A, Malami S, Albanesi EI, Dabed P, Molina C, López-Revilla R, Mandys V, González ME, Velasco J, Bravo IG, Quint W, Pawlita M, Muñoz N, de Sanjosé S, Xavier Bosch F, Ajayi OF, ICO International HPV in Head and Neck Cancer Study Group (2016). HPV involvement in head and neck cancers: comprehensive assessment of biomarkers in 3680 patients. J Natl Cancer Inst.

[5] Gooi Z, Chan JY, Fakhry C (2016). The epidemiology of the human papillomavirus related to oropharyngeal head and neck cancer. Laryngoscope..

[6] Ndiaye C, Alemany L, Diop Y, Ndiaye N, Diémé MJ, Tous S, Klaustermeier JE, Alejo M, Castellsagué X, Bosch FX, Trottier H, Sd S (2013). The role of human papillomavirus in head and neck cancer in Senegal. Infect Agents Cancer.

[7] Blumberg J, Monjane L, Prasad M, Carrilho C, Judson BL (2015). Investigation of the presence of HPV related oropharyngeal and oral tongue squamous cell carcinoma in Mozambique. Cancer Epidemiol.

[8] Oga EA, Schumaker LM, Alabi BS, Obaseki D, Umana A, Bassey IA, Ebughe G, Oluwole O, Akeredolu T, Adebamowo SN, Dakum P, Cullen K, Adebamowo CA (2016). Paucity of HPV-related head and neck cancers (HNC) in Nigeria. PLoS One.

[9] Chikandiwa A, Pisa PT, Sengayi M, Singh E, Delany-Moretlwe S (2019). Patterns and trends of HPV-related cancers other than cervix in South Africa from 1994-2013. Cancer Epidemiol.

[10] Bruni L, Barrionuevo-Rosas L, Albero G, Aldea M, Serrano B, Valencia S, Brotons M, Mena M, Cosano R, Muñoz J, Bosch FX, de Sanjosé S, Castellsagué X. ICO information Centre on HPV and Cancer (HPV information Centre). Human papillomavirus and related diseases in Gabon. Summary report 2016-02-26. [Data Accessed]. Available at: http://www.hpvcentre.net/statistics/reports/GAB.pdf (Last accessed July 21, 2016).

[11] Labouba I, Bertolus C, Koumakpayi HI, Belembaogo E, Miloundja J, Berthet N (2016). Impact of human papillomavirus on head and neck squamous cell cancers in Gabon. Infect Agent Cancer.

[12] Bruni L, Albero G, Serrano B, Mena M, Gómez D, Muñoz J, Bosch FX, de Sanjosé S. ICO/IARC Information Centre on HPV and Cancer (HPV Information Centre). Human Papillomavirus and Related Diseases in Central African Republic. Summary Report 10 December 2018. Available at: https://hpvcentre.net/statistics/reports/CAF.pdf. Accessed Mar 2019.

[13] Zehbe I, Wilander E (1996). Two consensus primer systems and nested polymerase chain reaction for human papillomavirus detection in cervical biopsies: a study of sensitivity. Hum Pathol.

[14] Kreimer AR, Clifford GM, Boyle P, Franceschi S (2005). Human papillomavirus types in head and neck squamous cell carcinomas worldwide: a systematic review. Cancer Epidemiol BiomarkersPrev.

[15] Weinberger PM, Merkley MA, Khichi SS, Lee JR, Psyrri A, Jackson LL (2010). Human papillomavirus-active head and neck Cancer and ethnic health disparities. Laryngoscope..

[16] Cai T, Di Vico T, Durante J, Tognarelli A, Bartoletti R (2018). Human papilloma virus and genitourinary cancers: a narrative review. Minerva Urol Nefrol.

[17] Alvarez-Aldana A, Martínez JW, Sepúlveda-Arias JC (2015). Comparison of five protocols to extract DNA from paraffin-embedded tissues for the detection of human papillomavirus. Pathol Res Pract.

